# A quasi-ballistic drain current, charge and capacitance model with positional carrier scattering dependency valid for symmetric DG MOSFETs in nanoscale regime

**DOI:** 10.1186/s40580-019-0189-y

**Published:** 2019-06-17

**Authors:** Vyas R. Murnal, C. Vijaya

**Affiliations:** Department of Electronics & Communication Engineering, SDM College of Engineering and Technology, Dharwad, Karnataka India

**Keywords:** Ballistic transport, Drift–diffusion, Quasi-ballistic, Scattering, SDG MOSFETs

## Abstract

This paper presents a physically valid quasi-ballistic drain current model applicable for nanoscale symmetric Double Gate (SDG) MOSFETs. The proposed drain current model includes both diffusive and ballistic transport phenomena. The model considers the important positional carrier scattering dependency effect near the source region described in terms of transmission and reflection co-efficients related to the scattering theory. The significance of carrier transport near the bottleneck source region is illustrated where the carriers diffuse into the channel at a relatively lower velocity before accelerating ballistically. The results obtained demonstrate carrier scattering dependency at the critical layer defined near the low field source region on the drain current characteristics. The proposed model partly evolves from Natori’s ballistic bulk MOSFET model that is modified accordingly to be valid for a symmetric Double Gate MOSFET in the nanoscale regime. Carrier degeneracy and Fermi–Dirac statistics are included in the work so as to justify the complete physicality of the model. The model is further extended and is shown to be continuous in terms of terminal charges and capacitances in all regions of operation. A comparative analysis is also done between the proposed quasi-ballistic model and a hypothetical complete ballistic device.

## Introduction

**Fig. 1:**
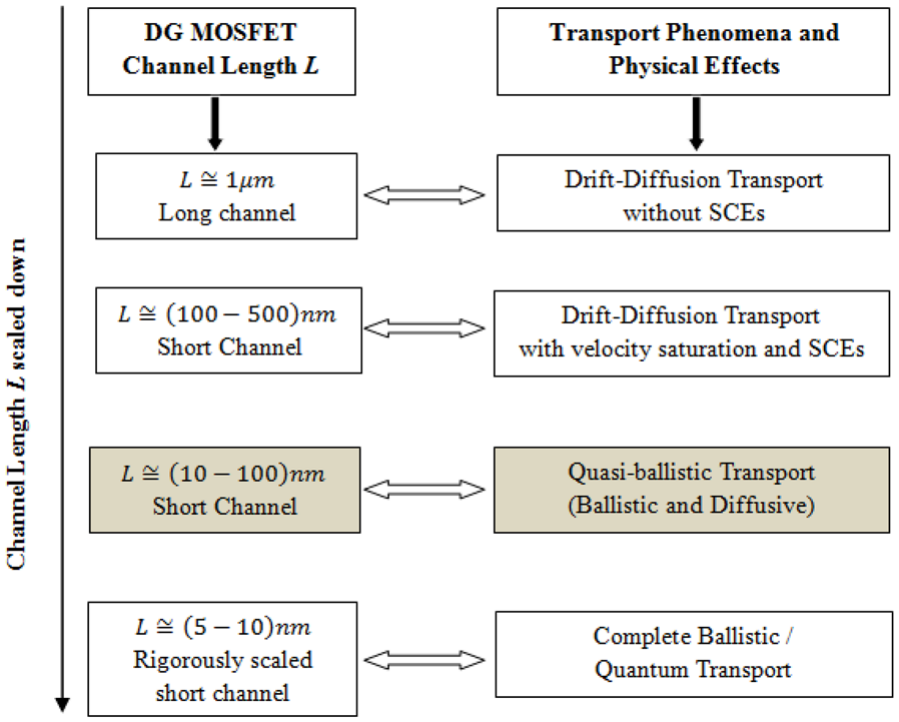
Different transport phenomena and physical effects arising when the channel length *L* of a DG MOSFET undergoes rigorous scaling. The grey shaded area is the core device channel length regime for the proposed work

Multiple gate MOSFETs have emerged as the most promising contenders [1] amongst various next generation semiconductor devices. A symmetric Double Gate (SDG) MOSFET is a variant of multigate MOSFETs, which is considered to be an ideal device that can be scaled beyond the bulk CMOS limit [2]. Mathematical modelling of MOSFETs has always been a challenging task whenever technology node and device generations change with respect to device channel length $L$. As the device channel lengths are scaled down from micrometer to nanometer regime, the carrier transport behaviour changes from drift–diffusive to ballistic [3]. In the nanoscale regime, initially the velocity saturation and then the source injection velocity limit become the major physical effects that limit the drain current. Figure 1 presents the various transport phenomena and occurrence of different physical effects when the device channel length $L$ undergoes rigorous scaling.

In nanoscale devices, the classical drift–diffusion equations [4] do not completely explain the device physics. The drift–diffusion transport fails to capture velocity overshoot and quantum mechanical tunnelling effects occurring in extremely scaled short channel devices. This may result in improper threshold voltage calculations. In such cases, the ballistic transport theory [5] must be considered and incorporated in device modelling, provided the quantum wave properties of electrons are ignored. If the channel length $L$ is much greater than the mean free path $\lambda$ of the carriers ($L > > \lambda$), then the transport mechanism is mainly drift-diffusive with significant amount of scattering. However, if the channel length $L$ is lesser than the mean free path $\lambda$ of the carriers ($L < < \lambda$) then the transport mechanism is primarily ballistic. Physically, $\lambda$ denotes the average distance between the scattering events. A complete ballistic MOSFET is a hypothetical device [6] without any scattering. Here, the drain current $I_{ds}$ is assumed to be independent of channel length, although the device will have finite ballistic channel resistance. Modern state of art nanoscale devices exhibit quasi-ballistic nature [7], because the maximum drain current is bounded by the rate at which carriers are injected from the source. This bottleneck condition suggests the need to include both diffusive and the ballistic transports appropriately while MOSFET modelling in the nanoscale regime.

This work presents a quasi-ballistic SDG MOSFET drain current model that evolves from Natori’s ballistic model. The proposed work considers the underlying physics of scattering effects in terms of transmission and reflection co-efficients evolving from the scattering theory [8]. The Natori’s model [9] proposes a fully ballistic bulk MOSFET model where the drain current is independent of the channel length $L$ and proportional to channel width $W$. The model also discusses about the maximum limit of current for a given MOSFET geometry. However, for a realistic quasi-ballistic case, this maximum current is reduced by the carrier flux injected from the source to the transport ballistic point in the channel. Although the proposed model partly evolves from Natori’s [9] ballistic bulk drain current model, the former work is completely different from the latter in terms of realistic physical behaviour. The proposed drain current model considers positional carrier scattering dependency in a nanoscale SDG MOSFET and includes both drift–diffusion and ballistic transport physics. The semi-classical approach used in this proposed work gives an intuitive analysis of the carrier scattering dependency characterized by the critical layer width $\delta ,$ near the low field source region on the drain current. The work includes carrier degeneracy effects and Fermi–Dirac statistics. The proposed model is found to be valid in the ballistic, diffusive limit and also in the quasi-ballistic regime. Further, the terminal charges and capacitances exhibit continuity in all regions of operation. A few analytical quasi-ballistic models [10, 11] for a DG MOSFET already do exist in the literature. To the best our knowledge these quasi-ballistic models neither consider positional carrier scattering dependency nor do they provide explicit solutions in terms of current and charges. The proposed work provides explicit solutions for the drain current, terminal charges and capacitances. These explicit expressions are highly preferred in compact modelling. Section 2 describes the physical analysis and mathematical background for the proposed work. Section 3 discusses the results obtained in accordance to the proposed model. Conclusion is done in Sect. 4.

## Model Description

The proposed model is described in two sub-sections (2.1 and 2.2). Sub-section 2.1 describes a fully ballistic nanoscale SDG MOSFET model that evolves from Natori’s ballistic model. In Sub-section 2.2, the proposed model of 2.1 is modified from complete ballistic to a realistic quasi-ballistic model by considering the carrier scattering dependency near the low field source region, in terms of transmission and reflection co-efficients as per the scattering theory.

**Fig. 2:**
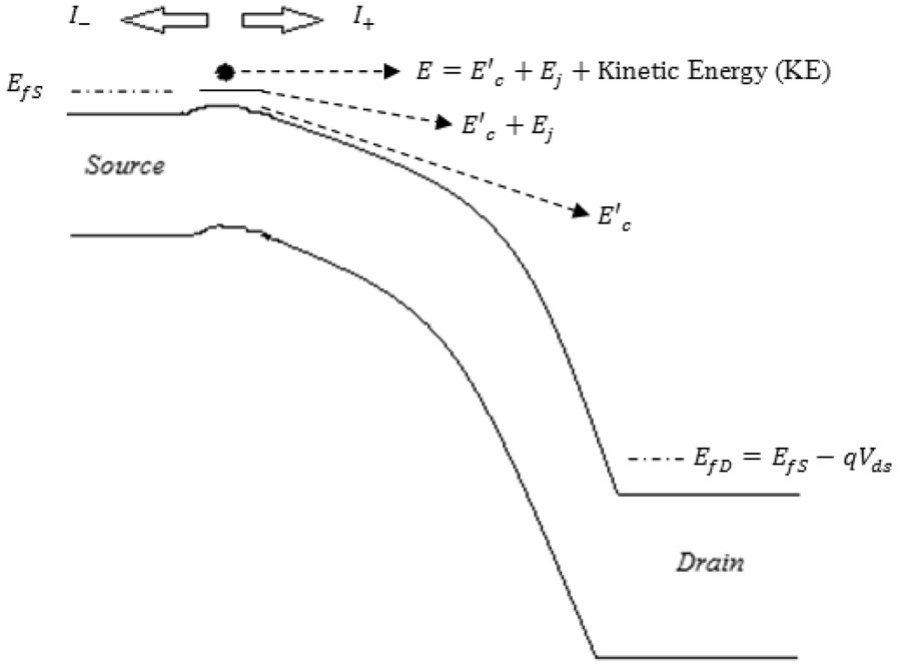
Schematic of a bulk nanoscale MOSFET band diagram under high-drain bias conditions [12]. For the proposed work of SDG MOSFET, a vertical mirror image of the above band diagram is to be considered due to bottom gate

2.1. Using the concept of flux theory, the net drain to source current in a bulk nanoscale MOSFET is expressed as

1 $$I_{ds} = I_{ + } - I_{ - }$$

where $I_{ + }$ is the left to right current component and $I_{ - }$ is the right to left current component as shown in Fig. 2. $E_{fS}$ and $E_{fD}$ in Fig. 2 represent source and drain Fermi levels respectively. The Fermi-levels of degenerately doped source and drain are indicated with dash-dot lines. The highest energy barrier appears to be near the source, where electrons with allowed discrete sub-bands populate. Carriers confined in an inversion layer occupy discrete sub- bands with a minimum energy $E_{j}$ above the conduction band $E^{\prime}_{c} .$ The probability of an electronic state being filled by an electron is given by the Fermi–Dirac distribution function

2 $$f_{D} \left( E \right) = \frac{1}{{1 + e^{{\frac{{\left( {E - E_{f} } \right)}}{kT}}} }}$$

where the parameter $E_{f}$ is the Fermi energy level. The energy level $E = (E'_{c} + E_{j} + {\text{KE}})$, is represented in Fig. 2. Under equilibrium conditions $I_{ds} = 0.$ For non-equilibrium conditions, the net drain to source current is expressed using one sub band approximation (lowest being j = 0 of unprimed valley) as

3 $$\begin{aligned} I_{{ds}} & = \, {\mathcal{G}}\left[ {F_{{1/2}} \left( {\frac{{E_{{fS}} - E_{c}^{\prime } - E_{0} }}{{kT}}} \right)} \right. \\ & \quad \left. - \, F_{{1/2}} \left( {\frac{{E_{{fS}} - E_{c}^{\prime } - E_{0} }}{{kT}} - \frac{{V_{{ds}} }}{{v_{T} }}} \right) \right]\\ \end{aligned}$$

where the parameter ${\mathcal{G}} = \frac{{8\sqrt {2m_{t} } qW(kT)^{3/2} }}{{h^{2} }}$ as in [12]. The Fermi–Dirac integral is then approximated as in [13] and given in (4) as

4 $$F_{{{\raise0.7ex\hbox{$1$} \!\mathord{\left/ {\vphantom {1 2}}\right.\kern-0pt} \!\lower0.7ex\hbox{$2$}}}} \left( {\frac{{E_{fS} - E^{\prime}_{c} - E_{0} }}{kT}} \right) \approx {\raise0.7ex\hbox{$2$} \!\mathord{\left/ {\vphantom {2 3}}\right.\kern-0pt} \!\lower0.7ex\hbox{$3$}}\left( {\frac{{E_{fS} - E^{\prime}_{c} - E_{0} }}{kT}} \right)^{{\frac{3}{2}}}$$

In (4) the expression in the bracket is given as

5 $$\begin{aligned} & \left( {\frac{{E_{{fS}} - E_{c}^{\prime } - E_{0} }}{{kT}}} \right) \hfill \\ & \quad = \ln \left[ {\frac{1}{2}\left\{ {\sqrt {\left( {e^{{\frac{{V_{{ds}} }}{{v_{T} }}}} - 1} \right)^{2} + 4\exp \left( g \right)} - 1 - e^{{\frac{{V_{{ds}} }}{{v_{T} }}}} } \right\}} \right] \hfill \\ \end{aligned}$$

Here, $g$ is another intermediary parameter given as

6 $$g = \left( {\frac{{h^{2} \varepsilon_{ox} \left( {V_{gs} - V_{t} } \right)}}{{4\pi qkTt_{ox} m_{t} }} + \frac{{V_{ds} }}{{v_{T} }}} \right)$$

In the above equations $h$ is the Plank’s constant, $\varepsilon_{ox}$ is the gate oxide permittivity, $V_{t}$ is the threshold voltage, $t_{ox}$ is the oxide layer thickness, thermal voltage $v_{T} = \frac{kT}{q}$ and $m_{t} = 0.19m_{0}$ where $m_{0}$ is the free electron mass. Substituting (5) and (6) in (4) and the resultant value in (3), the drain current for a bulk ballistic MOSFET is obtained that includes both Fermi–Dirac statistics and carrier degeneracy.

**Fig. 3:**
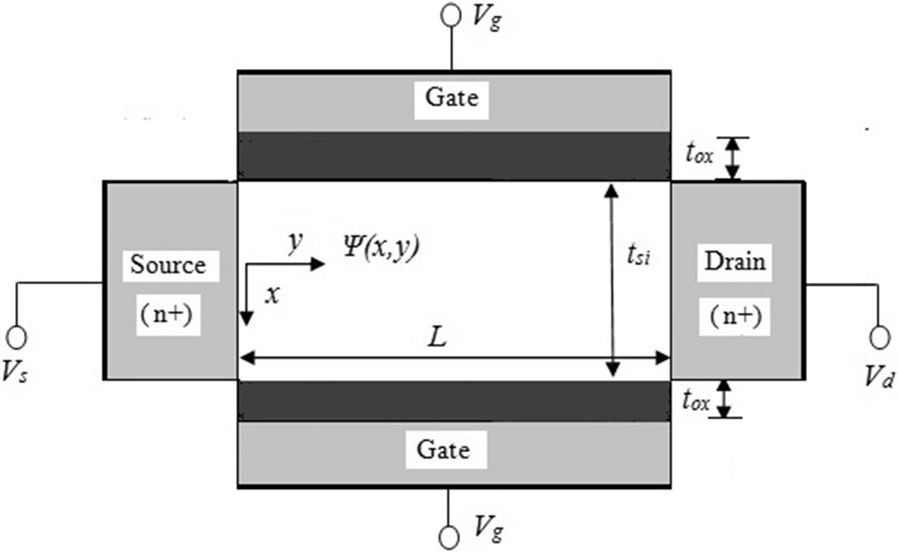
Schematic diagram of a symmetric Double Gate (SDG) MOSFET

Generally, symmetrical device structures simplify the mathematical analysis and steps involved in the modelling process. Hence, the device structure considered in the proposed work is a nanoscale symmetric Double Gate MOSFET (SDG) as mentioned in Fig. 3. In SDG MOSFETs, both gates have identical work function and hence switch together giving rise to two inversion channels (one at top of silicon film and the other at the bottom). The current and inversion charge capacitances are doubled in the double gate case. However, the prime advantage is in the ability of DG MOSFETs to scale to a shorter channel length well beyond bulk CMOS limit. Due to the aforementioned reasons, the drain current for a fully ballistic symmetric DG ballistic MOSFET is obtained by multiplying (3) with a factor of two that is mathematically expressed in (7) as

7 $$\begin{aligned} I_{ds} & = 2{\mathcal{G}}\left[ {F_{1/2} \left( {\frac{{E_{fS} - E^{\prime}_{c} - E_{0} }}{kT}} \right)} \right. \\ & \quad \left. -\, {F_{1/2} \left( {\frac{{E_{fS} - E^{\prime}_{c} - E_{0} }}{kT} - \frac{{V_{ds} }}{{v_{T} }}} \right)} \right] \end{aligned}$$

The silicon film in Fig. 3 is assumed to be lightly doped and fully depleted so that the discrete dopant fluctuations do not arise. The threshold voltage $V_{t}$ in (6) is determined as in [14] because it is completely dependent on the gate work function. In order to achieve lower threshold voltages, the proposed work considers n + polysilicon as gate material.

2.2. As per the ballistic transport theory, current needs to be evaluated at the top of the channel barrier. In a ballistic MOSFET, due to the ballistic injection process, velocity saturates at the top of the barrier where the electric field is zero. However, in a long channel device, velocity saturates near the drain due to scattering at the high electric field drain region. Equation (7) represents a fully ballistic DG MOSFET model that is independent of channel length when the mean free path of carriers exhibits the condition of ($\lambda \sim L$). The measured current values in [15] show that the modern nanoscale transistors of channel lengths in the range $\left( {10\,{\text{nm}} < L < 100\,{\text{nm}}} \right)$ are quasi-ballistic (i.e. approximately 20% to 50% of ballistic limit in the saturation part). The lesser current observed is due to the carrier scattering dependency observed near the low field source region in the semi-ballistic regime. As the channel length is scaled towards nanoscale regime, the proposed model satisfactorily includes the scattering effects in terms of transmission and reflection coefficients. Scattering equation in a nanoscale SDG MOSFET can be expressed from the elementary scattering theory in terms of transmission coefficient $(T_{C} )$ and reflection coefficient $(R_{C} )$ in (8) as

8 $$T_{C} + R_{C} = 1$$

where

9 $$T_{C} = \frac{\lambda }{\lambda + L }\quad {\text{and}} \quad R_{C} = \frac{L}{\lambda + L }$$

**Fig. 4:**
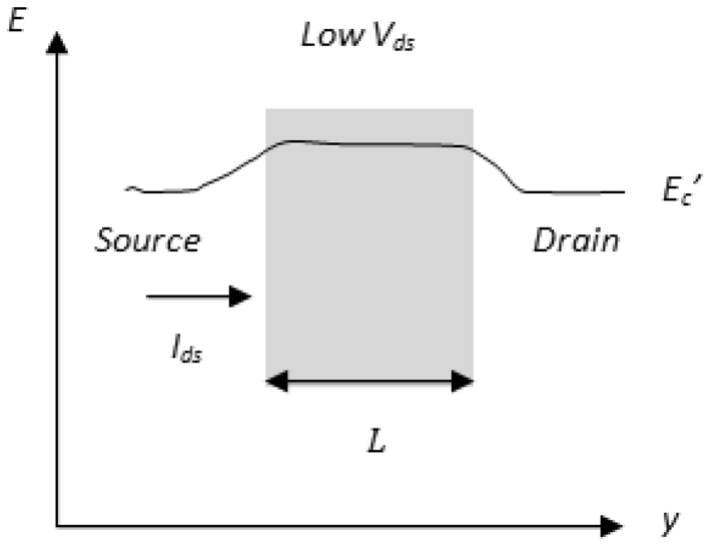
MOSFET band diagram under low drain bias conditions. Here ${\text{L}} >\uplambda$ condition is considered, so that scattering is uniform throughout the channel

**Fig. 5:**
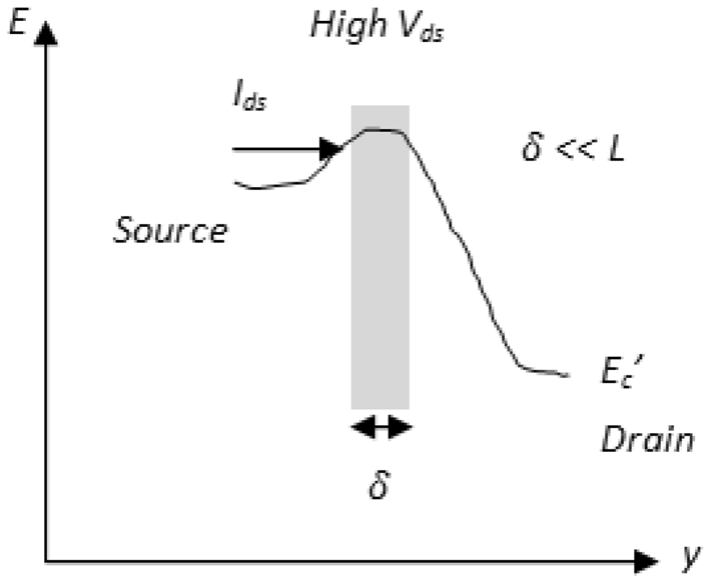
MOSFET band diagram under high drain bias conditions. Here $\updelta \ll {\text{L}}$ condition is considered. Carrier scattering depends on the critical layer width $\updelta ,$ near the low field source region

To avoid complexity, the proposed work assumes elastic scattering [16] and also considers that the average velocity of backscattered carriers is equal to that of injected carriers. Based on (8) and (9) a simple relation between diffusive and ballistic transport is given further. If $T_{C} = 0 \;{\text{and}}\;L \gg \lambda ,$ then the transport is drift-diffusive with significant scattering. Else, if $T_{C} = 1\; {\text{and}}\;L \ll \lambda ,$ then the transport is strongly ballistic and all injected carriers enter the drain. The mean free path $\lambda$ depends on effective mobility $\mu_{eff}$ of electrons (Appendix: Eq. 17). The effective mobility is calculated considering 2D electrostatics as in [17, 18] as a function of transverse electric field. A nanoscale DG MOSFET consists of a low field region near the source that is firmly controlled by gate voltage $V_{gs}$ and a high field region near the drain that is greatly controlled by drain voltage $V_{ds}$ as shown in Fig. 4. Here, the mean free path $\lambda$ and channel ${\text{length }}L$ are sufficient to determine transport with scattering for low drain bias. However, for high drain bias with the condition mentioned in Fig. 5, carrier scattering depends on the critical layer width $\delta ,$ near the low field source region. Scattering near the drain has a lesser effect on backscattering to the source. Hence, it is the scattering that occurs immediately after the critical layer $\delta$ near the source (as shown in Fig. 5) that matters for most of the transmission. This positional carrier scattering dependency near the low field small source region $\delta$ is responsible for quasi-ballistic nature of devices. Based on the preceding intuition and considering condition for the critical layer width near source as $\delta \ll L,$ the transmission co-efficient term in (9) is re-written by replacing *L* by $\delta$ and expressed in (10) as

10 $$T_{C} = \frac{\lambda }{\lambda + \delta }$$

Finally, using (10), the drain current equation in (7) is re-written and expressed in (11) as

11 $$\begin{aligned} I_{ds} &= 2{\mathcal{G}}T_{C} \left[ {F_{1/2} \left( {\frac{{E_{fS} - E^{\prime}_{c} - E_{0} }}{kT}} \right)} \right. \\ & \quad \left. - \, {F_{1/2} \left( {\frac{{E_{fS} - E^{\prime}_{c} - E_{0} }}{kT} - \frac{{qV_{ds} }}{kT}} \right)} \right] \end{aligned}$$

Thus, in very short channels, due to the quasi-ballistic transport, the carriers diffuse above the threshold region (top of the barrier near the source) unlike long channel where it is the drift current during high drain bias. This small bottleneck region that is usually lesser than the mean free path of the carriers, limits the current in a nanoscale device. After the critical layer width $\delta ,$ the ballistic transport starts to dominate. In Fig. 3 it is seen that due to the absence of body contacts, a SDG MOSFET can have only 3 terminals (with both gates tied). Let $Q_{g} ,Q_{d} \;{\text{and}}\;Q_{s}$ be the terminal charges associated with gate, drain and source respectively for the device shown in Fig. 3. The analytical expressions for the terminal charges applicable for a long channel SDG MOSFET are given in [19]. The terminal charges in the proposed model evolve from these analytical expressions and are modified accordingly to be applicable for nanoscale device lengths considering quasi-ballistic transport. Finally, the solutions for four capacitances (that are independent of each other), are also explicitly given as

12 $$C_{gd} = - \frac{{L^{2} g^{2}_{ds} }}{{\mu_{eff} I_{ds} }} + \frac{{Q_{g} g_{ds} }}{{I_{ds} }}$$

13 $$C_{sd} = \frac{{\left( {Q_{s} - Q_{d} } \right)g_{ds} }}{{I_{ds} }}$$

14 $$C_{dg} = - \frac{{L^{2} g^{2}_{ds} }}{{\mu_{eff} I_{ds} }} + \frac{{Q_{g} g_{ds} }}{{I_{ds} }} - \frac{{\left( {Q_{s} - Q_{d} } \right)g_{m} }}{{I_{ds} }}$$

15 $$C_{gs} = \frac{{L^{2} (g^{2}_{ds} + g_{m} )^{2} }}{{\mu_{eff} I_{ds} }} + \frac{{Q_{g} (g_{ds} + g_{m} )}}{{I_{ds} }}$$

where $g_{m}$ is the gate transconductance and $g_{ds}$ is drain-to-source conductance. The above expressions partially evolve from [20] and are modified accordingly so as to consider both diffusive and ballistic transport phenomena. The results in the next section illustrate and discuss the completeness of the model in terms of current, charges and capacitances.

## Results and discussions

This section provides results to the proposed quasi-ballistic model of Sect. 2. As mentioned in the previous section, the drain current in a fully ballistic device is independent of channel length $L$. However, realistic nanoscaled MOSFETs exhibit quasi-ballistic nature where drain current is quasi-independent of the channel length $L$. In such devices, $I_{ds }$ depends on the mean free path $\lambda$ and critical layer width $\delta$ near the low field source region.

**Fig. 6:**
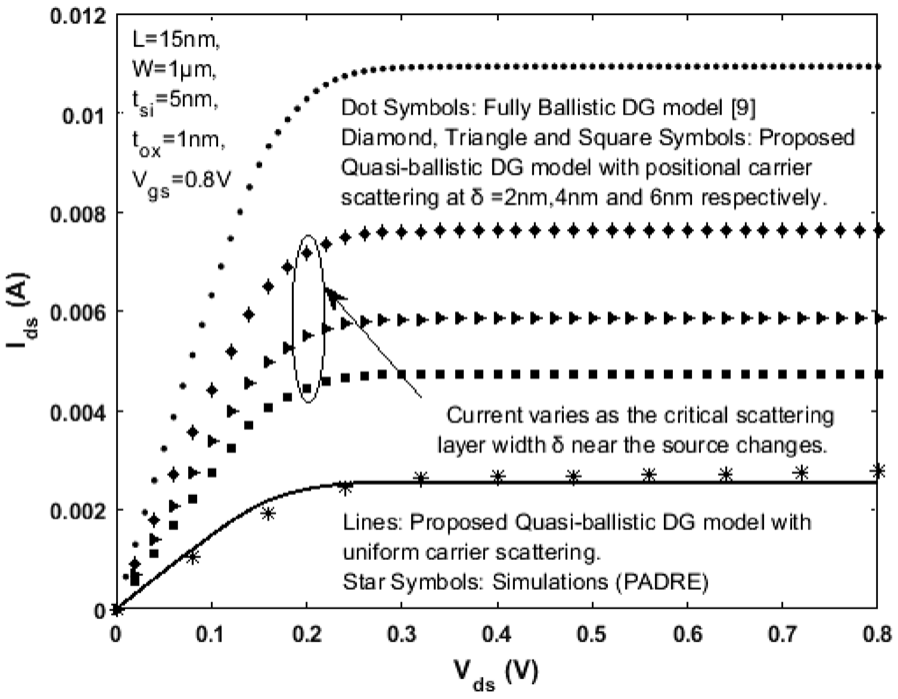
Variation of Drain current ${\text{I}}_{\text{ds }}$ as a function of ${\text{V}}_{\text{ds}}$ for ${\text{V}}_{\text{gs}}$ = 0.8 V in a SDG MOSFET. The proposed model is verified with the numerical simulation results obtained using MOSFet-PADRE tool [22]

**Fig. 7:**
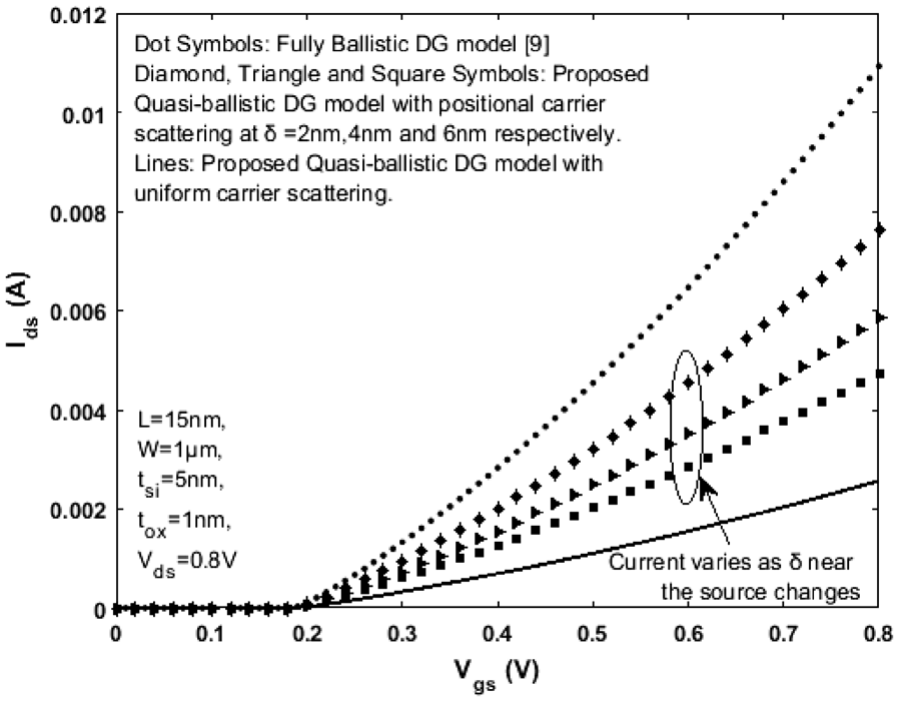
Variation of Drain current ${\text{I}}_{\text{ds }}$ as a function of ${\text{V}}_{\text{gs}}$ for ${\text{V}}_{\text{ds}}$ = 0.8 V in a SDG MOSFET

Here, width of the critical layer $\delta$ determines the scattering rate and magnitude of diffusive current present in the quasi-ballistic device. A rigorously scaled n-channel SDG MOSFET with the following values/specifications is considered for obtaining results: effective channel length *L* = 15 nm, width *W* = 1 μm, lightly doped Si film with doping density *N*_*a*_ = 1 x 10^12^ cm^−3^, bulk electron mobility *μ* = 300 cm^2^/v s, silicon film thickness *t*_*si*_ = 5 nm and oxide thickness *t*_*ox*_ = 1 nm. Effective mobility for electrons $\mu_{eff}$ is calculated as a function of surface potential $\psi_{s}$ and gate voltage and the same is used to obtain the mean free path $\lambda$. Using (Appendix: Eq. 17) mean free path $\lambda$ is obtained in the range of approximately 5–7 nm. The maximum thermal injection velocity $\nu_{therm}$ with which the electrons travel is calculated using (Appendix: Eq. 16) and is found to be approximately 2.2 × 10^7^ cm/s. Figures 6 and 7 represent the output and transfer characteristics respectively for the fully ballistic and the proposed quasi-ballistic model with positional scattering dependency. The fully ballistic drain current (Dots) obtained using (7) is significantly larger when compared with the proposed quasi-ballistic drain current (11) (Lines) with uniform scattering throughout the channel. Different critical layer widths near the low field source region are respectively chosen so that quasi-ballistic transport with carrier scattering is clearly observed. The positional carrier scattering dependency on the drain current near the low field source region is given by Diamond, Triangle and Square Symbols for critical layer width δ = 2 nm, 4 nm and 6 nm respectively. As the critical layer width $\delta$ is reduced for high drain bias, the corresponding semi-ballistic drain current rises and scales upwards towards the complete ballistic limit (represented by dot symbols in Fig. 6). More importantly, at the critical layer due to scattering, the carriers cannot diffuse faster than the thermal velocity $\nu_{therm}$ that actually limits the current. Beyond the critical layer, the transmission starts becoming ballistic. If $\delta$ increases beyond the mean free path $\lambda$ and approaches the dimensions of *L*, then the proposed drain current scales down towards the quasi-ballistic phase (i.e. without positional carrier scattering) representing uniform scattering throughout the channel (Lines in Fig. 6). These results justify that the top of the barrier (which is just near the low field source region) controls the transmission diffusively and it is the scattering at this critical region that matters the most and leads to the quasi-ballistic drain current observed in modern real nanoscale DG MOSFETs.

**Fig. 8:**
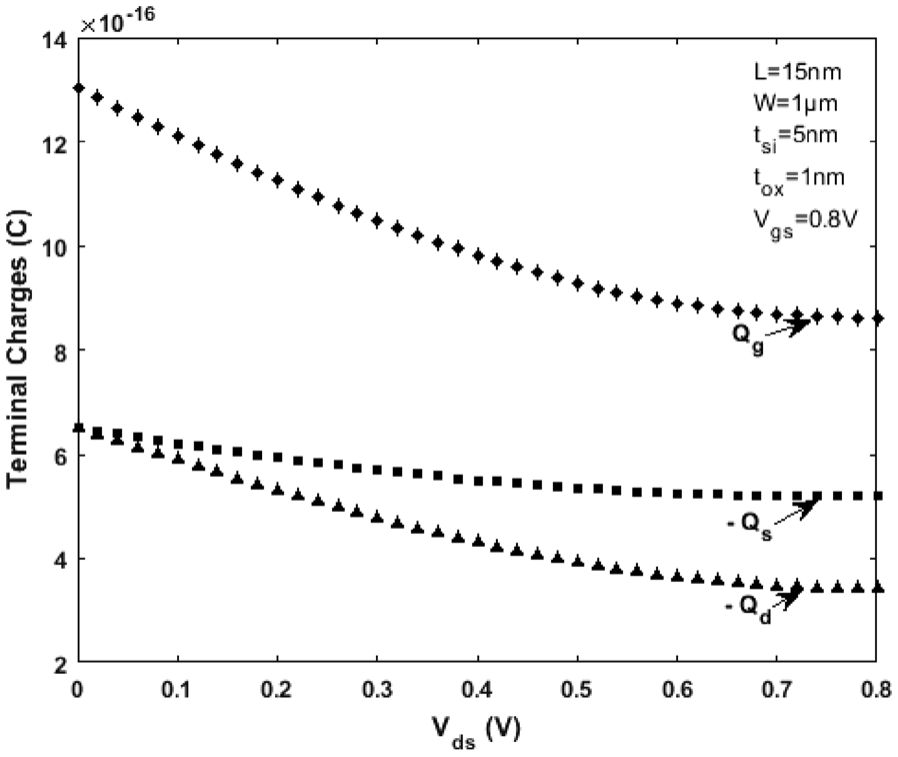
Variation of terminal charges as a function of drain voltage ${\text{V}}_{\text{ds}}$ represented by different symbols. ${\text{Q}}_{\text{g}} ,{\text{Q}}_{\text{d}} \;{\text{and}}\;{\text{Q}}_{\text{s}}$ represent gate, drain and source charge respectively

**Fig. 9:**
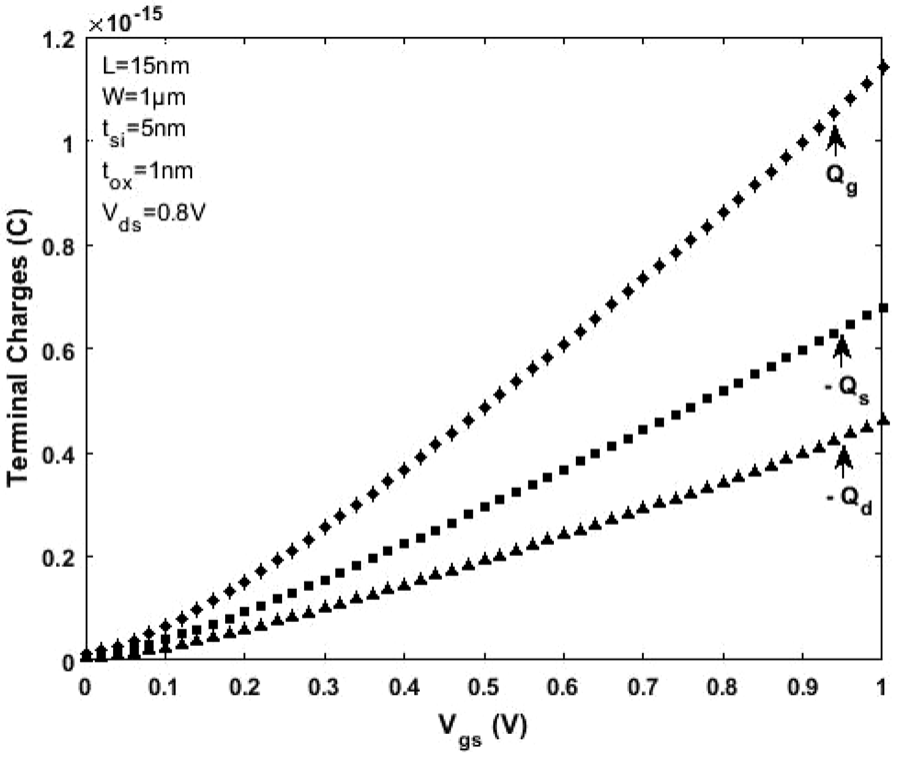
Variation of terminal charges as a function of gate voltage ${\text{V}}_{\text{gs}}$ represented by different symbols. ${\text{Q}}_{\text{g}} ,{\text{Q}}_{\text{d}} \;{\text{and}}\;{\text{Q}}_{\text{s}}$ represent gate, drain and source charge respectively

**Fig. 10:**
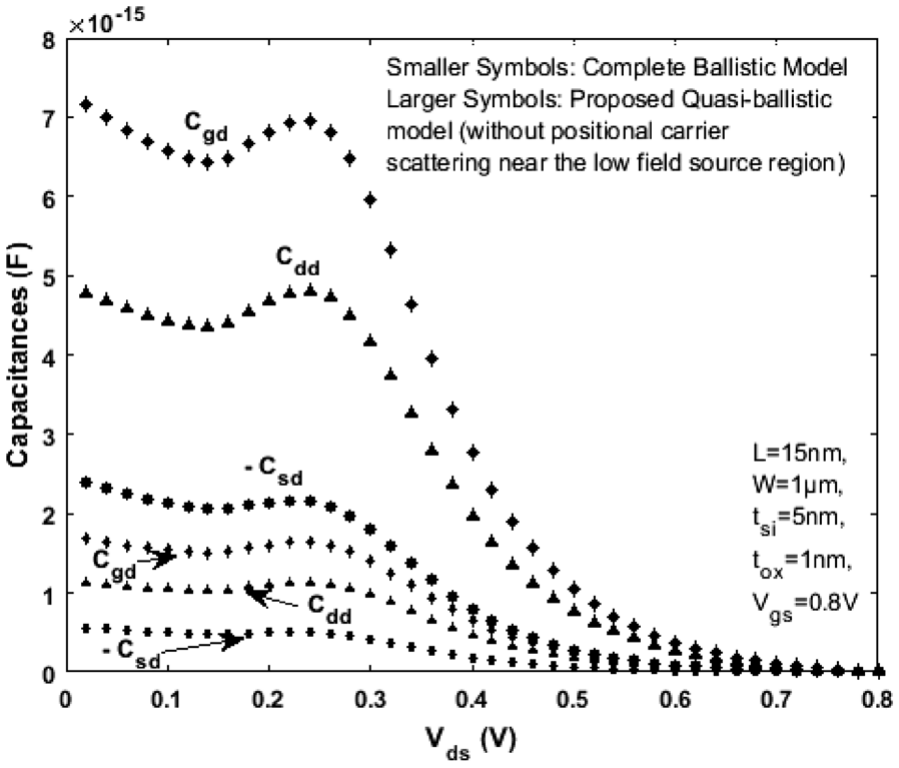
Variation of capacitances ${\text{C}}_{\text{gd}}$, ${\text{C}}_{\text{dd}}$, ${\text{C}}_{\text{sd}}$ as a function of drain voltage ${\text{V}}_{\text{ds}}$. Smaller size symbols are the capacitances as per complete Ballistic model (7) and larger size symbols are capacitances as per the proposed model (9) and (11) with uniform carrier scattering

**Fig. 11:**
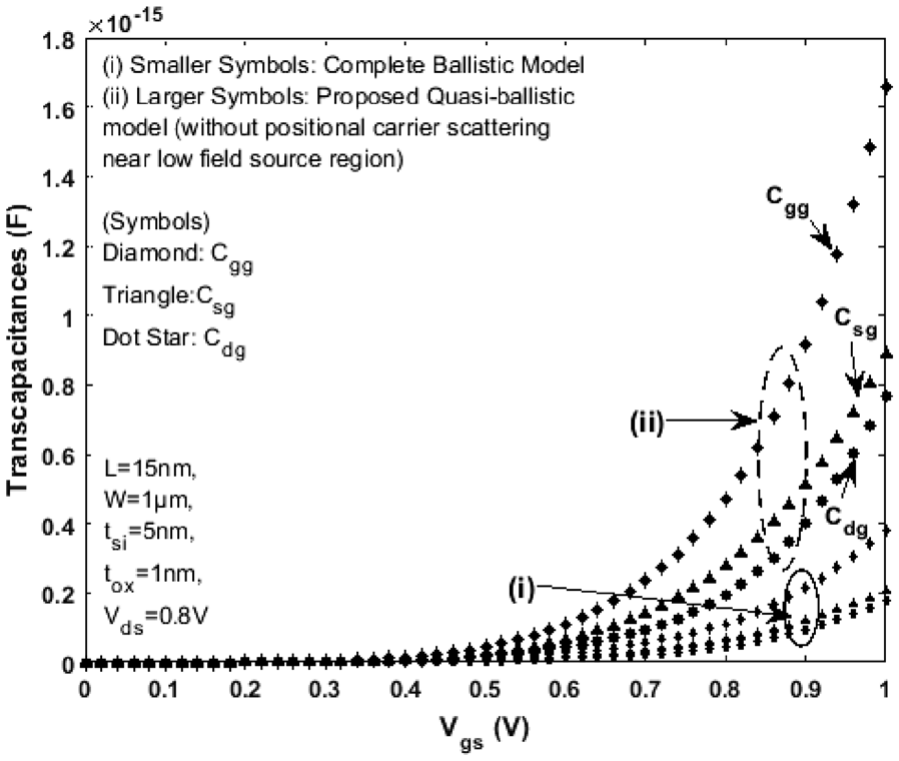
Variation of Transcapacitances ${\text{C}}_{\text{gg}}$, ${\text{C}}_{\text{sg}}$, ${\text{C}}_{\text{dg}}$ as a function of gate voltage ${\text{V}}_{\text{gs}}$. Smaller size symbols (i) are the capacitances as per complete Ballistic model (7) and larger size symbols (ii) are capacitances as per the proposed model (9) and (11) with uniform carrier scattering

Referring to the scale length theory for a DG MOSFET [21] and considering channel length L = 15 nm, the critical region width $\delta$ next to the top of barrier near the source region (as shown in Fig. 5) is approximately 2 to 5 nm (when the mean free path $\lambda$ is 6 to 7 nm). For a model to be complete, the drain current, terminal charges, conductance and capacitances must be continuous. Figures 8 and 9 illustrate the variation of terminal charges as a function of drain and gate voltages respectively for the proposed model. Further, the capacitances are calculated using (12), (13), (14) and (15). The variation of capacitances as a function of drain and gate voltages are shown in Figs. 10 and 11. In both Figs. 10 and 11 the proposed quasi-ballistic model using (9) and (11) gives larger capacitance values when compared with the ballistic model (7). If critical width parameter $\delta$ near the low field source region is considered as in (10) then the capacitance values with positional carrier scattering will lie between both models. It is observed that the terminal charges and capacitances calculated as per the proposed model exhibit excellent continuity in all regions of operation similar to the drain current. All the mathematical equations presented in the proposed work are coded and simulated using Matlab platform. The proposed model is verified with numerical simulation results obtained using MOSFet- PADRE tool [22]. To summarize, the proposed quasi-ballistic SDG MOSFET model is continuous in terms of drain current, charges and capacitances and hence can be considered in the next generation compact models.

## Conclusion

The work proposes a simple physics based quasi-ballistic drain current model applicable for nanoscale symmetric Double Gate (SDG) MOSFETs. The model considers positional carrier scattering effects near the source region described in terms of transmission and reflection co-efficients related to the scattering theory. The projected model is physical and includes both diffusive and ballistic transport phenomena considering the mean free path, channel length and critical region width near the low field source region. The proposed model is verified with the numerical simulation results obtained using MOSFet- PADRE tool. The drain current model exhibits excellent continuity in all regions of operation and hence may be considered suitably in the next generation circuit simulators for nanoscale modelling applications.

## Data Availability

The datasets used and/or analyzed during the current study are available from the corresponding author on reasonable request.

## Appendix

16 $${\text{Thermal injection velocity}}\,\left( {\nu_{therm} } \right) = \sqrt {\frac{2kT}{\pi m}}$$

where $m$ is effective mass of electron, $k$ is Boltzmann constant, $T = 300{\text{K}}$.

17 $${\text{Mean free path}}\, \left( \lambda \right) = \left( {\frac{{2v_{T} }}{{\nu_{therm} }} } \right)\mu_{eff}$$

## Abbreviations

DG: Double Gate
SDG: Symmetric Double Gate
